# Supplementary material for: Site-Specific Evolutionary Rate Shifts in HIV-1 and SIV
Source: Viruses. 2020 Nov 16;12(11):1312. doi: 10.3390/v12111312 (PMC7696578; doi:10.3390/v12111312)
Supplement: Supplementary file 1 [file viruses-12-01312-s001.zip › Supplementary information.pdf]

Supplementary Material

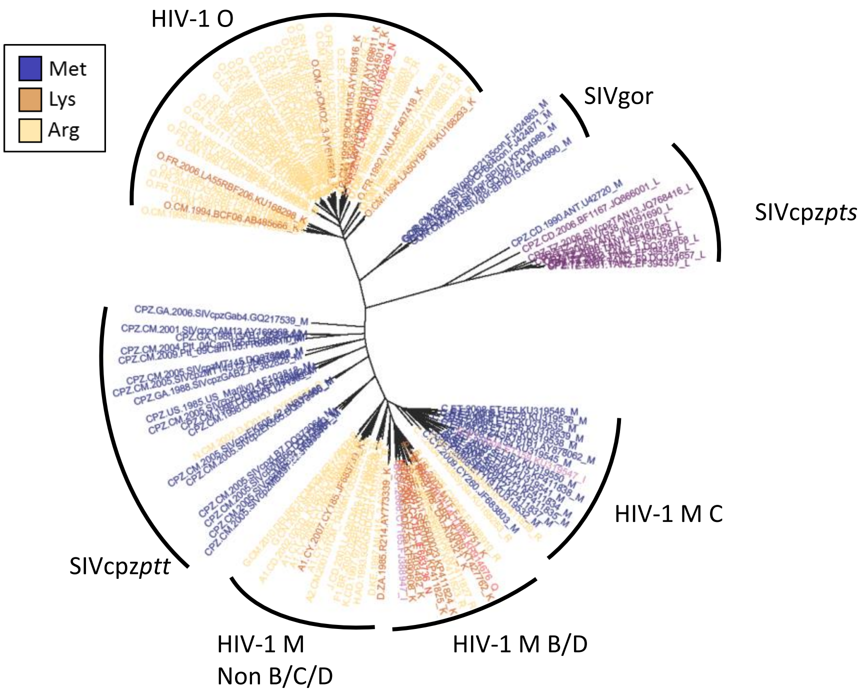

Fig. S1. Residues distribution for Gag_{30} along the HIV-1/SIV phylogeny. This position exhibits a pattern more similar to "content shift" than to "rate shift".

Table S1. Maximum log-likelihood (LL) values for the analysis of the nine HIV-1/SIVcpz/SIVgor proteins under the rate shift and null models.

Protein	Rate Shift Model LL	Null Model LL	2ΔLL	P-value (χ^2_3)
**Gag**	-29,478.3	-29,671.4	386	$<10^{-50}$
**Pol**	-40,813.1	-41,097.2	568	$<10^{-100}$
**Vif**	-12,677.6	-12,756.3	157	$<10^{-30}$
**Vpr**	-5,328.1	-5,355.76	55	$<10^{-10}$
**Tat**	-8,918.23	-8,947.61	59	$<10^{-10}$
**Rev**	-12,662	-12,733.6	143	$<10^{-30}$
**Vpu**	-10,548.7	-10,664.1	231	$<10^{-50}$
**Env**	-92,347.6	-93,028.8	1362	$<10^{-100}$
**Nef**	-15,611.4	-15,804.1	385	$<10^{-50}$

Table S2. Rate shifts as *percentage from total rate shifts for prominent branches*, for rate decelerating sites (upper) and rate accelerating sites (lower), colored by intensity. Data presented in this table is not normalized by protein length.

DEC<br>Branch/protein	Env	Gag	Pol	Rev	Tat	Nef	Vif	Vpr	Vpu
**Group M**	39%	21%	21%	4%	0%	0%	0%	0%	14%
**Group O**	43%	11%	11%	20%	0%	4%	2%	2%	7%
**SIVgor+P+O**	21%	7%	29%	0%	0%	29%	14%	0%	0%
**SIVcpz*pts***	26%	15%	7%	0%	0%	22%	11%	4%	15%
ACC<br>Branch/protein	**Env**	**Gag**	**Pol**	**Rev**	**Tat**	**Nef**	**Vif**	**Vpr**	**Vpu**
**Group M**	43%	22%	4%	0%	4%	13%	4%	4%	4%
**Group O**	55%	18%	12%	6%	0%	4%	4%	0%	2%
**SIVgor+P+O**	40%	0%	0%	0%	0%	40%	0%	10%	10%
**SIVcpz*pts***	35%	10%	10%	0%	0%	23%	3%	6%	13%

Table S3. Rate deceleration events that may be related to previously reported species-specific adaptation events.

Protein	Clade	Adapting sites	Potential adaptation	Functional domain near adaptive sites	Supporting literature	Description
**Nef**	SIVcpz*ptt*	157	Anti-tetherin	163, 169	(Gotz et al. 2012)	HIV-1 adapted to chimpanzee reverted Nef_{163} and Nef_{169} positions to regain anti-tetherin activity
**Nef**	HIV-1 O + SIVgor	177	Anti-tetherin	165-173 (C-loop)	(Mack 2017)	O-Nef counteracts tetherin using its C-loop
**Vif**	HIV-1 O + SIVgor	73, 167	Anti-A3G	69-72 (A3G binding domain), 161-169 (Oligomerization domain)	(Pery et al. 2009; Letko et al. 2013; Feng et al. 2014; Letko et al. 2015)	Gorilla-A3G differs from human-A3G in the Vif-recognized domain
**Vif**	HIV-1 O	127	Anti-A3G	120-124 (Cullin5 interacting domain)	(Feng et al. 2014)	Gorilla-A3G differs from human-A3G in the Vif-recognized domain.
**Gag**	HIV-1 M	30	Unknown	30	(Wain et al. 2007)	This site is accepted as an adaptation marker to human of HIV-1 groups M and O. Our analysis identified a rate deceleration but inconclusive for which clade rate deceleration happened (Supplementary Fig. 2)

A3G=APOBEC3G;

File S1.

Phylogenetic tree and sequence alignments used for identifying rate shifting sites. The zip file contains all nine alignments and constructed phylogeny used for identifying rate shifting sites.

File S2.

HIV/SIV sites identified as rate shifting. Positions are provided in HXB2 reference sequence coordinates. Branch number field refer to the branch number outputted by RASER (Penn et al. 2008); for ease of reading we provide branch labels for most branches.

File S3.

HIV/SIV sites identified as rate shifting when group M contains many more sequenced than group O. Positions are provided in HXB2 reference sequence coordinates. Branch number field refer to the branch number outputted by RASER (Penn et al. 2008); for ease of reading we provide branch labels for most branches.

References

Feng Y, Baig TT, Love RP and Chelico L (2014). "Suppression of APOBEC3-mediated restriction of HIV-1 by Vif." Front Microbiol 5: 450.

Gotz N et al. (2012). "Reacquisition of Nef-Mediated Tetherin Antagonism in a Single In Vivo Passage of HIV-1 through Its Original Chimpanzee Host." Cell Host & Microbe 12(3): 373-380.

Letko M et al. (2015). "Identification of the HIV-1 Vif and Human APOBEC3G Protein Interface." Cell Rep 13(9): 1789-1799.

Letko M et al. (2013). "Vif Proteins from Diverse Primate Lentiviral Lineages Use the Same Binding Site in APOBEC3G." Journal of Virology 87(21): 11861-11871.

Mack K (2017). Counteraction of the antiviral factor tetherin by HIV-1 group O, Universität Ulm.

Penn O et al. (2008). "Evolutionary modeling of rate shifts reveals specificity determinants in HIV-1 subtypes." PLoS Comput Biol 4(11): e1000214.

Pery E, Rajendran KS, Brazier AJ and Gabuzda D (2009). "Regulation of APOBEC3 proteins by a novel YXXL motif in human immunodeficiency virus type 1 Vif and simian immunodeficiency virus SIVagm Vif." J. Virol. 83(5): 2374-2381.

Wain LV et al. (2007). "Adaptation of HIV-1 to its human host." Mol. Biol. Evol. 24(8): 1853-1860.
